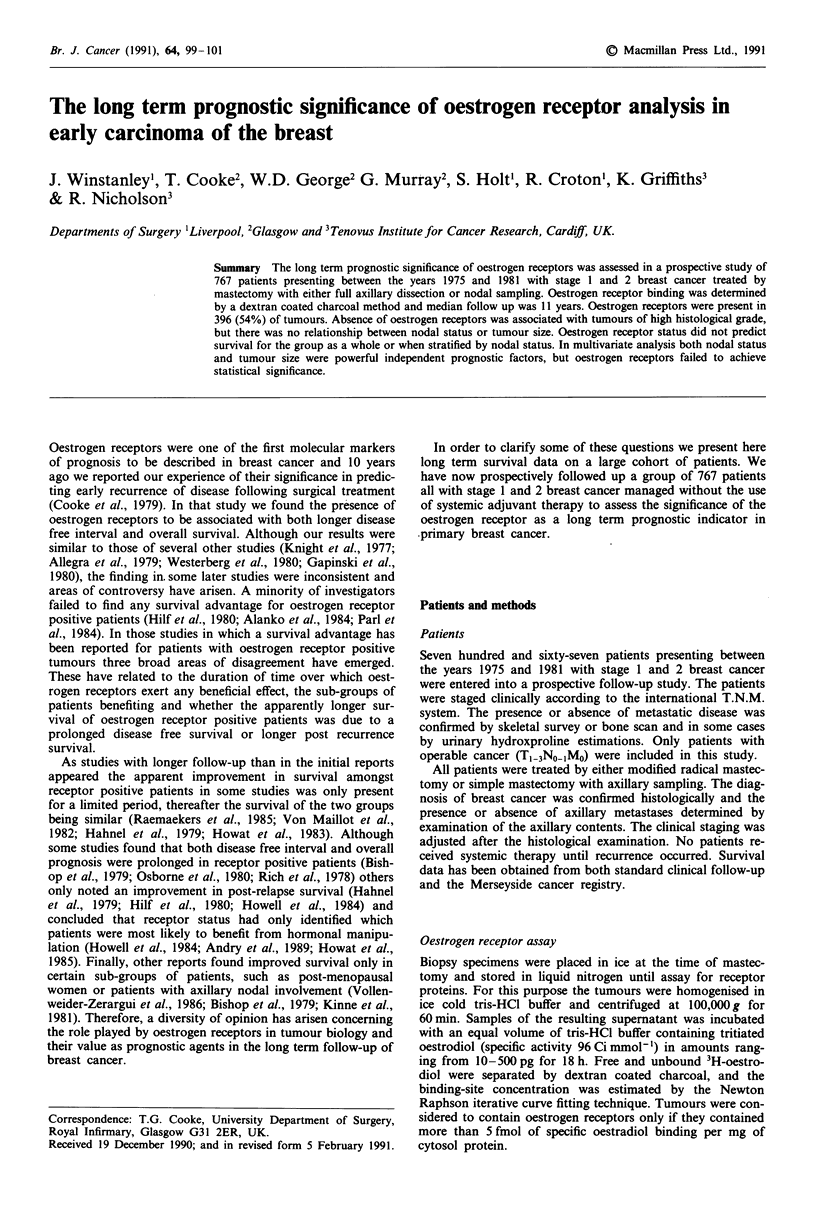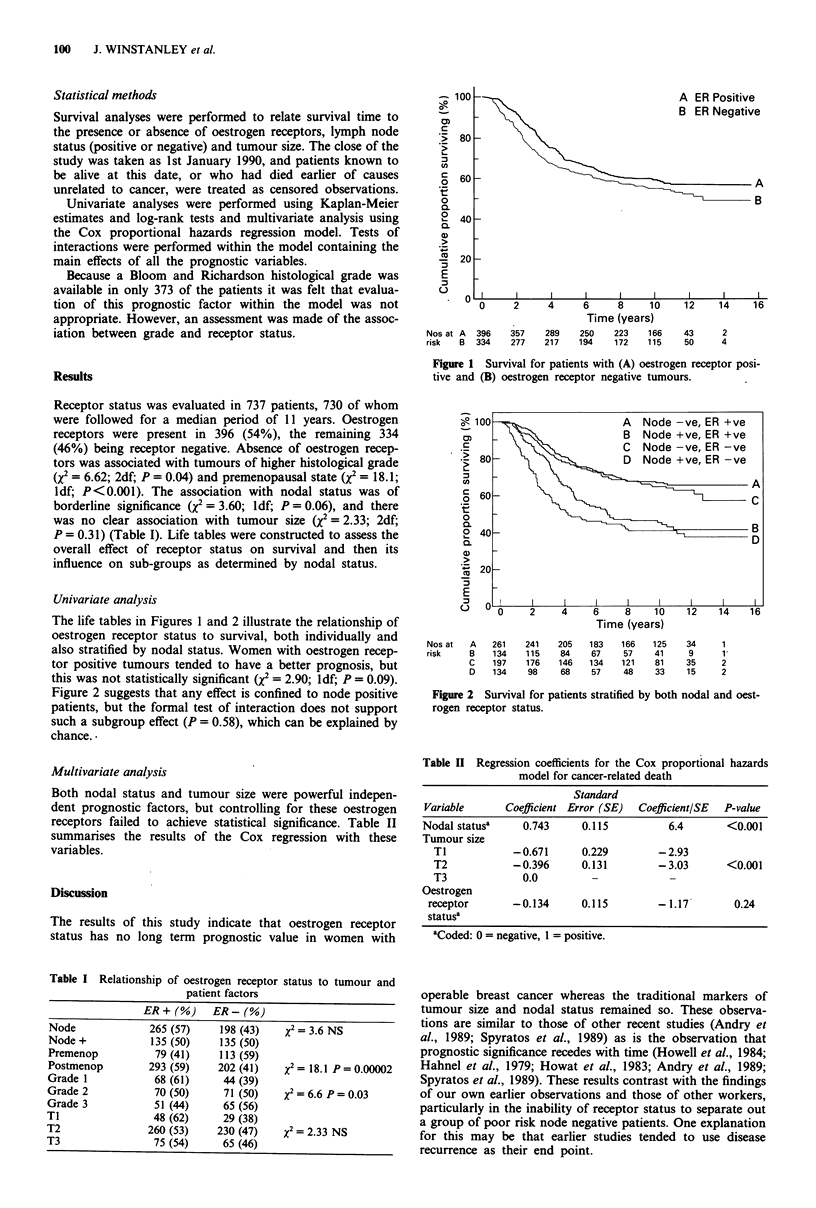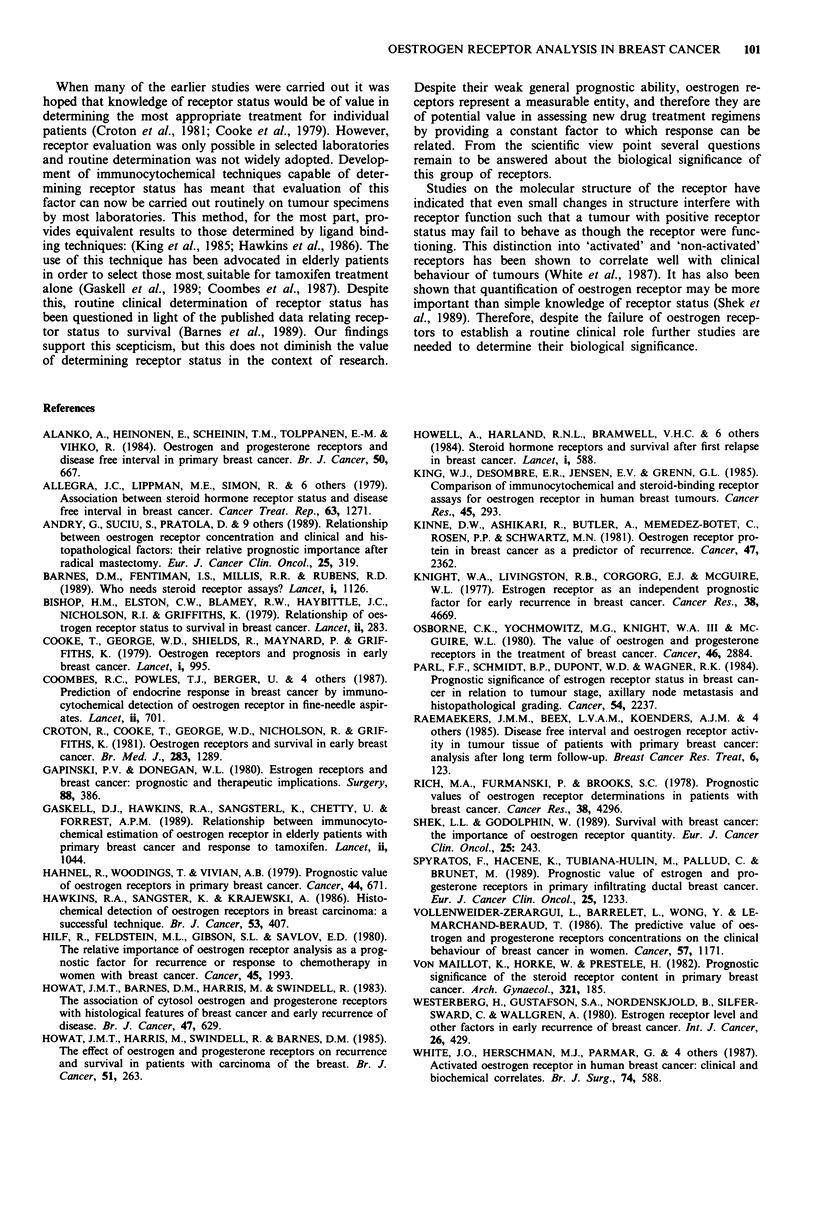# The long term prognostic significance of oestrogen receptor analysis in early carcinoma of the breast.

**DOI:** 10.1038/bjc.1991.249

**Published:** 1991-07

**Authors:** J. Winstanley, T. Cooke, W. D. George, G. Murray, S. Holt, R. Croton, K. Griffiths, R. Nicholson

**Affiliations:** Department of Surgery Liverpool, Institute for Cancer Research, Cardiff, U.K.

## Abstract

The long term prognostic significance of oestrogen receptors was assessed in a prospective study of 767 patients presenting between the years 1975 and 1981 with stage 1 and 2 breast cancer treated by mastectomy with either full axillary dissection or nodal sampling. Oestrogen receptor binding was determined by a dextran coated charcoal method and median follow up was 11 years. Oestrogen receptors were present in 396 (54%) of tumours. Absence of oestrogen receptors was associated with tumours of high histological grade, but there was no relationship between nodal status or tumour size. Oestrogen receptor status did not predict survival for the group as a whole or when stratified by nodal status. In multivariate analysis both nodal status and tumour size were powerful independent prognostic factors, but oestrogen receptors failed to achieve statistical significance.


Br. J. Cancer (1991), 64, 99-101 © Macmillan Press Ltd., 1991

### The long term prognostic significance of oestrogen receptor analysis in early carcinoma of the breast

J. Winstanley[1], T. Cooke[2], W.D. George[2] G. Murray[2], S. Holt[1], R. Croton[1], K. Griffiths[3] & R. Nicholson[3]

*Departments of Surgery [1]Liverpool, [2]Glasgow and [3]Tenovus Institute for Cancer Research, Cardiff, UK.*

**Summary** The long term prognostic significance of oestrogen receptors was assessed in a prospective study of 767 patients presenting between the years 1975 and 1981 with stage 1 and 2 breast cancer treated by mastectomy with either full axillary dissection or nodal sampling. Oestrogen receptor binding was determined by a dextran coated charcoal method and median follow up was 11 years. Oestrogen receptors were present in 396 (54%) of tumours. Absence of oestrogen receptors was associated with tumours of high histological grade, but there was no relationship between nodal status or tumour size. Oestrogen receptor status did not predict survival for the group as a whole or when stratified by nodal status. In multivariate analysis both nodal status and tumour size were powerful independent prognostic factors, but oestrogen receptors failed to achieve statistical significance.

Oestrogen receptors were one of the first molecular markers of prognosis to be described in breast cancer and 10 years ago we reported our experience of their significance in predicting early recurrence of disease following surgical treatment (Cooke *et al.*, 1979). In that study we found the presence of oestrogen receptors to be associated with both longer disease free interval and overall survival. Although our results were similar to those of several other studies (Knight *et al.*, 1977; Allegra *et al.*, 1979; Westerberg *et al.*, 1980; Gapinski *et al.*, 1980), the finding in some later studies were inconsistent and areas of controversy have arisen. A minority of investigators failed to find any survival advantage for oestrogen receptor positive patients (Hilf *et al.*, 1980; Alanko *et al.*, 1984; Parl *et al.*, 1984). In those studies in which a survival advantage has been reported for patients with oestrogen receptor positive tumours three broad areas of disagreement have emerged. These have related to the duration of time over which oestrogen receptors exert any beneficial effect, the sub-groups of patients benefiting and whether the apparently longer survival of oestrogen receptor positive patients was due to a prolonged disease free survival or longer post recurrence survival.

As studies with longer follow-up than in the initial reports appeared the apparent improvement in survival amongst receptor positive patients in some studies was only present for a limited period, thereafter the survival of the two groups being similar (Raemaekers *et al.*, 1985; Von Maillot *et al.*, 1982; Hahnel *et al.*, 1979; Howat *et al.*, 1983). Although some studies found that both disease free interval and overall prognosis were prolonged in receptor positive patients (Bishop *et al.*, 1979; Osborne *et al.*, 1980; Rich *et al.*, 1978) others only noted an improvement in post-relapse survival (Hahnel *et al.*, 1979; Hilf *et al.*, 1980; Howell *et al.*, 1984) and concluded that receptor status had only identified which patients were most likely to benefit from hormonal manipulation (Howell *et al.*, 1984; Andry *et al.*, 1989; Howat *et al.*, 1985). Finally, other reports found improved survival only in certain sub-groups of patients, such as post-menopausal women or patients with axillary nodal involvement (Vollenweider-Zerargui *et al.*, 1986; Bishop *et al.*, 1979; Kinne *et al.*, 1981). Therefore, a diversity of opinion has arisen concerning the role played by oestrogen receptors in tumour biology and their value as prognostic agents in the long term follow-up of breast cancer.

Correspondence: T.G. Cooke, University Department of Surgery, Royal Infirmary, Glasgow G31 2ER, UK.
Received 19 December 1990; and in revised form 5 February 1991.

In order to clarify some of these questions we present here long term survival data on a large cohort of patients. We have now prospectively followed up a group of 767 patients all with stage 1 and 2 breast cancer managed without the use of systemic adjuvant therapy to assess the significance of the oestrogen receptor as a long term prognostic indicator in primary breast cancer.

### Patients and methods

### *Patients*

Seven hundred and sixty-seven patients presenting between the years 1975 and 1981 with stage 1 and 2 breast cancer were entered into a prospective follow-up study. The patients were staged clinically according to the international T.N.M. system. The presence or absence of metastatic disease was confirmed by skeletal survey or bone scan and in some cases by urinary hydroxproline estimations. Only patients with operable cancer ($T_{1-3}N_{0-1}M_0$) were included in this study.

All patients were treated by either modified radical mastectomy or simple mastectomy with axillary sampling. The diagnosis of breast cancer was confirmed histologically and the presence or absence of axillary metastases determined by examination of the axillary contents. The clinical staging was adjusted after the histological examination. No patients received systemic therapy until recurrence occurred. Survival data has been obtained from both standard clinical follow-up and the Merseyside cancer registry.

### *Oestrogen receptor assay*

Biopsy specimens were placed in ice at the time of mastectomy and stored in liquid nitrogen until assay for receptor proteins. For this purpose the tumours were homogenised in ice cold tris-HCl buffer and centrifuged at 100,000 *g* for 60 min. Samples of the resulting supernatant was incubated with an equal volume of tris-HCl buffer containing tritiated oestrodiol (specific activity 96 Ci mmol$^{-1}$) in amounts ranging from 10-500 pg for 18 h. Free and unbound $^3$H-oestrodiol were separated by dextran coated charcoal, and the binding-site concentration was estimated by the Newton Raphson iterative curve fitting technique. Tumours were considered to contain oestrogen receptors only if they contained more than 5 fmol of specific oestradiol binding per mg of cytosol protein.

### *Statistical methods*

Survival analyses were performed to relate survival time to the presence or absence of oestrogen receptors, lymph node status (positive or negative) and tumour size. The close of the study was taken as 1st January 1990, and patients known to be alive at this date, or who had died earlier of causes unrelated to cancer, were treated as censored observations.

Univariate analyses were performed using Kaplan-Meier estimates and log-rank tests and multivariate analysis using the Cox proportional hazards regression model. Tests of interactions were performed within the model containing the main effects of all the prognostic variables.

Because a Bloom and Richardson histological grade was available in only 373 of the patients it was felt that evaluation of this prognostic factor within the model was not appropriate. However, an assessment was made of the association between grade and receptor status.

### Results

Receptor status was evaluated in 737 patients, 730 of whom were followed for a median period of 11 years. Oestrogen receptors were present in 396 (54%), the remaining 334 (46%) being receptor negative. Absence of oestrogen receptors was associated with tumours of higher histological grade ($\chi^2 = 6.62$; 2df; $P = 0.04$) and premenopausal state ($\chi^2 = 18.1$; 1df; $P < 0.001$). The association with nodal status was of borderline significance ($\chi^2 = 3.60$; 1df; $P = 0.06$), and there was no clear association with tumour size ($\chi^2 = 2.33$; 2df; $P = 0.31$) (Table I). Life tables were constructed to assess the overall effect of receptor status on survival and then its influence on sub-groups as determined by nodal status.

### *Univariate analysis*

The life tables in Figures 1 and 2 illustrate the relationship of oestrogen receptor status to survival, both individually and also stratified by nodal status. Women with oestrogen receptor positive tumours tended to have a better prognosis, but this was not statistically significant ($\chi^2 = 2.90$; 1df; $P = 0.09$). Figure 2 suggests that any effect is confined to node positive patients, but the formal test of interaction does not support such a subgroup effect ($P = 0.58$), which can be explained by chance.

### *Multivariate analysis*

Both nodal status and tumour size were powerful independent prognostic factors, but controlling for these oestrogen receptors failed to achieve statistical significance. Table II summarises the results of the Cox regression with these variables.

### Discussion

The results of this study indicate that oestrogen receptor status has no long term prognostic value in women with operable breast cancer whereas the traditional markers of tumour size and nodal status remained so. These observations are similar to those of other recent studies (Andry *et al.*, 1989; Spyratos *et al.*, 1989) as is the observation that prognostic significance recedes with time (Howell *et al.*, 1984; Hahnel *et al.*, 1979; Howat *et al.*, 1983; Andry *et al.*, 1989; Spyratos *et al.*, 1989). These results contrast with the findings of our own earlier observations and those of other workers, particularly in the inability of receptor status to separate out a group of poor risk node negative patients. One explanation for this may be that earlier studies tended to use disease recurrence as their end point.

**Figure 1** Survival for patients with (A) oestrogen receptor positive and (B) oestrogen receptor negative tumours.

**Figure 2** Survival for patients stratified by both nodal and oestrogen receptor status.

**Table II** Regression coefficients for the Cox proportional hazards model for cancer-related death

| *Variable* | *Coefficient* | *Standard Error (SE)* | *Coefficient/SE* | *P-value* |
|---|---|---|---|---|
| Nodal status[a] | 0.743 | 0.115 | 6.4 | <0.001 |
| Tumour size | | | | |
| T1 | −0.671 | 0.229 | −2.93 | |
| T2 | −0.396 | 0.131 | −3.03 | <0.001 |
| T3 | 0.0 | – | – | |
| Oestrogen receptor status[a] | −0.134 | 0.115 | −1.17 | 0.24 |

[a]Coded: 0 = negative, 1 = positive.

**Table I** Relationship of oestrogen receptor status to tumour and patient factors

| | *ER + (%)* | *ER − (%)* | |
|---|---|---|---|
| Node | 265 (57) | 198 (43) | $\chi^2 = 3.6$ NS |
| Node + | 135 (50) | 135 (50) | |
| Premenop | 79 (41) | 113 (59) | |
| Postmenop | 293 (59) | 202 (41) | $\chi^2 = 18.1$ $P = 0.00002$ |
| Grade 1 | 68 (61) | 44 (39) | |
| Grade 2 | 70 (50) | 71 (50) | $\chi^2 = 6.6$ $P = 0.03$ |
| Grade 3 | 51 (44) | 65 (56) | |
| T1 | 48 (62) | 29 (38) | |
| T2 | 260 (53) | 230 (47) | $\chi^2 = 2.33$ NS |
| T3 | 75 (54) | 65 (46) | |

When many of the earlier studies were carried out it was hoped that knowledge of receptor status would be of value in determining the most appropriate treatment for individual patients (Croton *et al*., 1981; Cooke *et al*., 1979). However, receptor evaluation was only possible in selected laboratories and routine determination was not widely adopted. Development of immunocytochemical techniques capable of determining receptor status has meant that evaluation of this factor can now be carried out routinely on tumour specimens by most laboratories. This method, for the most part, provides equivalent results to those determined by ligand binding techniques: (King *et al*., 1985; Hawkins *et al*., 1986). The use of this technique has been advocated in elderly patients in order to select those most suitable for tamoxifen treatment alone (Gaskell *et al*., 1989; Coombes *et al*., 1987). Despite this, routine clinical determination of receptor status has been questioned in light of the published data relating receptor status to survival (Barnes *et al*., 1989). Our findings support this scepticism, but this does not diminish the value of determining receptor status in the context of research. Despite their weak general prognostic ability, oestrogen receptors represent a measurable entity, and therefore they are of potential value in assessing new drug treatment regimens by providing a constant factor to which response can be related. From the scientific view point several questions remain to be answered about the biological significance of this group of receptors.

Studies on the molecular structure of the receptor have indicated that even small changes in structure interfere with receptor function such that a tumour with positive receptor status may fail to behave as though the receptor were functioning. This distinction into 'activated' and 'non-activated' receptors has been shown to correlate well with clinical behaviour of tumours (White *et al*., 1987). It has also been shown that quantification of oestrogen receptor may be more important than simple knowledge of receptor status (Shek *et al*., 1989). Therefore, despite the failure of oestrogen receptors to establish a routine clinical role further studies are needed to determine their biological significance.

### References

ALANKO, A., HEINONEN, E., SCHEININ, T.M., TOLPPANEN, E.-M. & VIHKO, R. (1984). Oestrogen and progesterone receptors and disease free interval in primary breast cancer. *Br. J. Cancer*, **50**, 667.

ALLEGRA, J.C., LIPPMAN, M.E., SIMON, R. & 6 others (1979). Association between steroid hormone receptor status and disease free interval in breast cancer. *Cancer Treat. Rep*., **63**, 1271.

ANDRY, G., SUCIU, S., PRATOLA, D. & 9 others (1989). Relationship between oestrogen receptor concentration and clinical and histopathological factors: their relative prognostic importance after radical mastectomy. *Eur. J. Cancer Clin. Oncol*., **25**, 319.

BARNES, D.M., FENTIMAN, I.S., MILLIS, R.R. & RUBENS, R.D. (1989). Who needs steroid receptor assays? *Lancet*, **i**, 1126.

BISHOP, H.M., ELSTON, C.W., BLAMEY, R.W., HAYBITTLE, J.C., NICHOLSON, R.I. & GRIFFITHS, K. (1979). Relationship of oestrogen receptor status to survival in breast cancer. *Lancet*, **ii**, 283.

COOKE, T., GEORGE, W.D., SHIELDS, R., MAYNARD, P. & GRIFFITHS, K. (1979). Oestrogen receptors and prognosis in early breast cancer. *Lancet*, **i**, 995.

COOMBES, R.C., POWLES, T.J., BERGER, U. & 4 others (1987). Prediction of endocrine response in breast cancer by immunocytochemical detection of oestrogen receptor in fine-needle aspirates. *Lancet*, **ii**, 701.

CROTON, R., COOKE, T., GEORGE, W.D., NICHOLSON, R. & GRIFFITHS, K. (1981). Oestrogen receptors and survival in early breast cancer. *Br. Med. J*., **283**, 1289.

GAPINSKI, P.V. & DONEGAN, W.L. (1980). Estrogen receptors and breast cancer: prognostic and therapeutic implications. *Surgery*, **88**, 386.

GASKELL, D.J., HAWKINS, R.A., SANGSTERL, K., CHETTY, U. & FORREST, A.P.M. (1989). Relationship between immunocytochemical estimation of oestrogen receptor in elderly patients with primary breast cancer and response to tamoxifen. *Lancet*, **ii**, 1044.

HAHNEL, R., WOODINGS, T. & VIVIAN, A.B. (1979). Prognostic value of oestrogen receptors in primary breast cancer. *Cancer*, **44**, 671.

HAWKINS, R.A., SANGSTER, K. & KRAJEWSKI, A. (1986). Histochemical detection of oestrogen receptors in breast carcinoma: a successful technique. *Br. J. Cancer*, **53**, 407.

HILF, R., FELDSTEIN, M.L., GIBSON, S.L. & SAVLOV, E.D. (1980). The relative importance of oestrogen receptor analysis as a prognostic factor for recurrence or response to chemotherapy in women with breast cancer. *Cancer*, **45**, 1993.

HOWAT, J.M.T., BARNES, D.M., HARRIS, M. & SWINDELL, R. (1983). The association of cytosol oestrogen and progesterone receptors with histological features of breast cancer and early recurrence of disease. *Br. J. Cancer*, **47**, 629.

HOWAT, J.M.T., HARRIS, M., SWINDELL, R. & BARNES, D.M. (1985). The effect of oestrogen and progesterone receptors on recurrence and survival in patients with carcinoma of the breast. *Br. J. Cancer*, **51**, 263.

HOWELL, A., HARLAND, R.N.L., BRAMWELL, V.H.C. & 6 others (1984). Steroid hormone receptors and survival after first relapse in breast cancer. *Lancet*, **i**, 588.

KING, W.J., DESOMBRE, E.R., JENSEN, E.V. & GRENN, G.L. (1985). Comparison of immunocytochemical and steroid-binding receptor assays for oestrogen receptor in human breast tumours. *Cancer Res*., **45**, 293.

KINNE, D.W., ASHIKARI, R., BUTLER, A., MEMEDEZ-BOTET, C., ROSEN, P.P. & SCHWARTZ, M.N. (1981). Oestrogen receptor protein in breast cancer as a predictor of recurrence. *Cancer*, **47**, 2362.

KNIGHT, W.A., LIVINGSTON, R.B., CORGORG, E.J. & MCGUIRE, W.L. (1977). Estrogen receptor as an independent prognostic factor for early recurrence in breast cancer. *Cancer Res*., **38**, 4669.

OSBORNE, C.K., YOCHMOWITZ, M.G., KNIGHT, W.A. III & MCGUIRE, W.L. (1980). The value of oestrogen and progesterone receptors in the treatment of breast cancer. *Cancer*, **46**, 2884.

PARL, F.F., SCHMIDT, B.P., DUPONT, W.D. & WAGNER, R.K. (1984). Prognostic significance of estrogen receptor status in breast cancer in relation to tumour stage, axillary node metastasis and histopathological grading. *Cancer*, **54**, 2237.

RAEMAEKERS, J.M.M., BEEX, L.V.A.M., KOENDERS, A.J.M. & 4 others (1985). Disease free interval and oestrogen receptor activity in tumour tissue of patients with primary breast cancer: analysis after long term follow-up. *Breast Cancer Res. Treat*, **6**, 123.

RICH, M.A., FURMANSKI, P. & BROOKS, S.C. (1978). Prognostic values of oestrogen receptor determinations in patients with breast cancer. *Cancer Res*., **38**, 4296.

SHEK, L.L. & GODOLPHIN, W. (1989). Survival with breast cancer: the importance of oestrogen receptor quantity. *Eur. J. Cancer Clin. Oncol*., **25**: 243.

SPYRATOS, F., HACENE, K., TUBIANA-HULIN, M., PALLUD, C. & BRUNET, M. (1989). Prognostic value of estrogen and progesterone receptors in primary infiltrating ductal breast cancer. *Eur. J. Cancer Clin. Oncol*., **25**, 1233.

VOLLENWEIDER-ZERARGUI, L., BARRELET, L., WONG, Y. & LEMARCHAND-BERAUD, T. (1986). The predictive value of oestrogen and progesterone receptors concentrations on the clinical behaviour of breast cancer in women. *Cancer*, **57**, 1171.

VON MAILLOT, K., HORKE, W. & PRESTELE, H. (1982). Prognostic significance of the steroid receptor content in primary breast cancer. *Arch. Gynaecol*., **321**, 185.

WESTERBERG, H., GUSTAFSON, S.A., NORDENSKJOLD, B., SILFERSWARD, C. & WALLGREN, A. (1980). Estrogen receptor level and other factors in early recurrence of breast cancer. *Int. J. Cancer*, **26**, 429.

WHITE, J.O., HERSCHMAN, M.J., PARMAR, G. & 4 others (1987). Activated oestrogen receptor in human breast cancer: clinical and biochemical correlates. *Br. J. Surg*., **74**, 588.